# Non-Technical Skills Bingo—a game to facilitate the learning of complex concepts

**DOI:** 10.1186/s41077-016-0024-z

**Published:** 2016-07-22

**Authors:** Peter Dieckmann, Ronnie Glavin, Rikke Malene Hartvigsen Grønholm Jepsen, Ralf Krage

**Affiliations:** 1 Copenhagen Academy for Medical Education and Simulation (CAMES), Center for Human Resources, Capital Region of Denmark, Herlev Hospital, 25th floor, Herlev Ringvej 75, 2370 Herlev, Denmark; 2 Scottish Centre for Simulation and Clinical Human Factors, Larbert, UK; 3 VU University Medical Center, Simulation Center ADAM, Amsterdam, The Netherlands

**Keywords:** Concept learning, Cognitive psychology, Non-technical skills, Gaming

## Abstract

Acquiring the concepts of non-technical skills (NTS) beyond a superficial level is a challenge for healthcare professionals and simulation faculty. Current simulation-based approaches to teach NTS are challenged when learners have to master NTS concepts, clinically challenging situations, and simulation as a complex technique. The combination of all three aspects might overwhelm learners. To facilitate the deeper comprehension of NTS concepts, we describe an innovative video-based game, the Non-Technical Skills (NTS) Bingo. Participants get NTS Bingo cards that show five NTS elements each. While observing (non-medical) video clips, they try to find examples for the elements on their cards, typically observable behaviours that match a given element. After the video, participants “defend” their solution in a discussion with the game leader and other players. This discussion and the reflection aim to deepen the processing of the NTS concepts. We provide practical guidance for the conduct of NTS Bingo, including a selection of usable video clips and tips for the facilitated discussion after a clip. We use NTS in anaesthesia as example and provide guidance on how to adapt NTS Bingo to other disciplines. NTS Bingo is based on theoretical considerations on concept learning, which we describe to support the rationale for its conduct.

**Electronic supplementary material:**

The online version of this article (doi:10.1186/s41077-016-0024-z) contains supplementary material, which is available to authorized users.

## Introduction

Non-technical skills (NTS) are an integrated part of the abilities that healthcare professionals require to optimally diagnose and treat patients under the complex conditions of healthcare. NTS in healthcare have been defined as “the cognitive and social skills that complement technical skills and medical knowledge in task performance” [1]. The use of NTS aims to improve patient outcome through both an increase in patient safety and operational efficiency [2–4]. NTS are applied widely in conceptual and empirical papers, including those pertaining to simulation in healthcare [5–10]. There are several frameworks to describe NTS, for example, crisis resource management (CRM) [10] or the TeamSTEPPS framework [11]. The term “non-technical skills” is under debate [12]; however, due to its current widespread use, we have opted to use it here for the purposes of this paper.

**Fig. 1:**
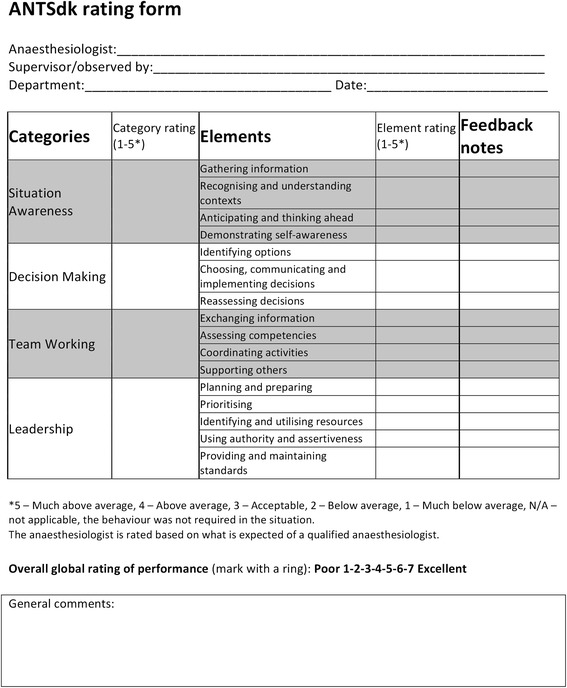
ANTSdk overview

We refer to the Danish variant of the Anaesthetists’ Non-Technical Skills system, ANTSdk [6], in its English translation (Fig. 1). ANTSdk comprises the four categories “situation awareness”, “decision making”, “leadership”, and “team work”. Each category has a number of underlying elements. Each element is in turn described by numerous behavioural markers, examples of positive and negative behaviours. ANTSdk will be used to illustrate the game-based exercise, called NTS Bingo. It is possible to adapt the NTS Bingo to other frameworks, disciplines, and professions. We use the discussion of ANTSdk and the application of NTS Bingo to anaesthesia as an example.

Several societies for anaesthesiology, intensive care medicine or medical education have included NTS training as crucial for patient safety [13–15]. The European Resuscitation Council [16] also includes NTS into their recent resuscitation guidelines. NTS are increasingly becoming part of medical licencing [8]. In Denmark, NTS sub-dimensions are an integrated part of the so-called competence cards that anaesthesiologists are required to collect as part of the portfolio on which their licencing is based. The Danish and Dutch systems, for example, build on the CanMeds Roles [17]. These roles are “medical expert”, “communicator”, “leader”, “health advocate”, “collaborator”, “scholar”, and “professional”. NTS can naturally be integrated into these roles.

One of the main functions of NTS frameworks is to stimulate discussion around how healthcare professionals can behave more safely and effectively. All healthcare professionals use NTS, even if they are unaware of them. The NTS frameworks are intended to help their users reflect on their existing NTS with the intention of being able to retain what works well and improve those actions that are not effective. Such reflection requires that learners understand how to apply the different ANTSdk categories and elements to their behaviours in different clinical contexts.

Learning to use, teach, and assess NTS are all complex endeavours. A new language underpinned by new concepts is being introduced. Concepts are more than terms [18]. Concepts structure what we perceive and how we orient ourselves in the world. Their importance is also emphasized in many debriefing models used in healthcare simulation, for example, the mental frames that should be investigated during debriefings [19]. Often we are not directly aware of the concepts we hold—we take many things for granted (the laws of gravity for example). NTS Bingo tries to stimulate reflection on the concepts that learners hold by asking participants to apply the concepts in a context that participants are not familiar with. The terms used in ANTSdk can seem deceptively simple and may create the illusion of a deep conceptual understanding, whereas in fact users might only have processed the terms on a superficial level, without forming robust concepts underlying the terms [20, 21]. The terms used to describe ANTSdk categories and ANTSdk elements often overlap in the complexity of the clinical world, which can present challenges when trying to get a grasp of the underlying concepts. NTS Bingo was created with the aim of supporting the formation of NTS concepts and improving the understanding of NTS terms.

We suggest that NTS Bingo can support simulation practice by helping simulation facilitators to improve their own understanding of NTS. This would be beneficial for a scenario design, as facilitators might then be able to design NTS challenges more systematically into scenarios [22, 23]. For debriefing, such an improved understanding of the NTS concepts will help simulation facilitators to guide the discussions towards NTS concepts [24–26]. An improved understanding of NTS will help simulation course participants to perform better during the simulation and also promote the development of better care in the clinical setting. A shared understanding of NTS between facilitators and participants should facilitate the discussions and mutual understanding which in turn should lead to more effective and deeper reflection during debriefing [27, 28].

One key feature of ANTSdk relevant in this context is that the elements in the ANTSdk categories “leadership” and “teamworking” tend to be more accessible to direct observation, whereas the ANTSdk elements in “situation awareness” and “decision making” tend to require more inference about mental processes based on observed behaviour. NTS Bingo could contribute to making this distinction clearer to simulation facilitators.

We did not formally evaluate NTS Bingo but conducted approximately 30 sessions during conferences and faculty development programmes as proof of concept. Oral feedback for those sessions was positive. Several groups working with NTS in simulation have adopted NTS into their practice.

## Theoretical considerations

In this section, we provide theoretical considerations to explain why we consider NTS Bingo an effective strategy to learn about NTS. We focus our considerations on “concept learning”, a study area within cognitive psychology [29]. We first discuss how “concepts” are discussed in the literature. We then describe how the acquisition of concepts can be supported.

Concepts provide the knowledge upon which a person is able to do a categorization, which is the process of recognizing what things, actions, or events belong together [29]. Concept learning is more than merely knowing a certain expression (e.g. recalling the steps in an algorithm). Grasping the concept, for example, that the pitch in the saturation tone in a patient monitor changes with oxygen levels in the blood of the monitored patient allows a person to distinguish patients with good oxygen saturation from patients with oxygenation problems. For the sake of simplicity, we will use the word “entity” to summarize things, actions, or events. Thus, concepts are used to mentally categorize entities as examples or non-examples of a mental category. The ANTSdk categories and elements are examples of such mental categories. They can be used to categorize human action in the context of anaesthesia in relation to NTS. In order to clearly distinguish the two senses of the word “category” in this paper, we will use *ANTSdk categories* to refer to the words in the ANTSdk system. We will use *mental category* to describe the cognitive structure of facilitators and participants.

There are different types of concepts [30]:

- *Concrete concepts* allow for the categorization of entities based on their physical characteristics (e.g. “a laryngoscope”, “a person”).
- *Defined concepts* are based on more abstract features of entities and relations between entities for the categorization of entities (e.g. “an airway management device”, “a treatment regime”).
- *Complex concepts* are composed of connected sub-elements and also often include an action perspective during the categorization of entities (e.g. “difficult airway management”, “treating anaphylactic shock”).

The more familiar a person is with a given concept, the better this person will be able to correctly classify entities as an example or non-example of a mental category—in a variety of contexts and where the entities do not have clear features to serve as a basis for classification (e.g. an atypical case of a disease, unfamiliar communication techniques) [30].

Concepts are not solely verbal [31]. The mere word is not enough to describe the concept (e.g. “teamwork”, “situation awareness”, “leadership”, “love”, or “reality”). Grasping a concept involves also “feelings, beliefs, and other affective components” [31, 32]. This is a part of the explanation why several people might use a certain expression fluidly in a conversation in shared understanding and yet, when asked to describe their concept underlying the expression, they might describe it very differently.

A strategy to support concept learning has been described [33]. In a first step, relevant features of a concept can be discussed, for example, by stating a definition. In a second step, learners can be presented with entities that are clear examples of a mental category (e.g. actions that are clear examples for one of the ANTSdk elements). These entities should be very clearly examples of the ANTSdk element and should not contain many irrelevant or accidental features. In a next step, learners can be presented with non-examples for a mental category (e.g. actions that clearly do not belong into the ANTSdk element under discussion). Then learners can gradually be introduced to more ambiguous entities that are not clearly an example or non-example of the ANTSdk element under discussion. Finally, concepts can be applied in new contexts to support their generalization (e.g. using the ANTSdk element to categorize non-medical entities as examples or non-examples for the element). During generalization, the learner becomes clearer about the difference between the essential features of a concept and any arbitrary features. Generalization of a concept is needed to also categorize atypical entities correctly. Consider the ANTSdk element “identifying options” as an example. In many cases, it would be irrelevant whether decision options are communicated orally or in writing, as long as the different options are considered.

Typical challenges in concept learning are misconceptions, overgeneralizations, and undergeneralizations: Misconceptions describe instances where learners form an understanding of a concept that is not in line with accepted definitions [31]. In undergeneralization, learners would categorize an actual example entity wrongly as non-example for a mental category. In overgeneralization, learners would categorize an actual non-example entity wrongly as example for a mental category [33].

## Applying the theoretical considerations to teaching NTS

Consider the ANTSdk element “gathering information”. In a teaching session, this can be defined as “vigilantly and systematically seeking out information about the situation” [6]. Then learners could discuss entities from their own practice area that they see as clear examples of “gathering information”. Participants could then discuss what entities that they see as non-examples for “gathering information”. Then participants could be asked to describe examples and non-examples for “gathering information” from a variety of contexts, for example, from their own professional past or private life. Finally, the group could discuss which entities might be difficult to classify as either examples or non-examples for “gathering information” to explore the borders of the concept. Learners could discuss for “gathering information” that considering *all* sources of information is not helpful because of a danger of information overload. Ignoring important information sources, on the other hand, might provide an incomplete picture of the situation and that may result in wrong treatment decisions.

How to concretely implement such a concept teaching sequence depends on the learners and their previous knowledge. The video examples chosen for NTS Bingo can be based on their anticipated clarity as examples or non-examples for ANTSdk categories and ANTSdk elements. Some of the clips we suggest below contain clear examples for some ANTSdk elements but not others. They thus provide good training opportunities to categorize entities as example or non-example of ANTSdk elements.

## Playing NTS Bingo

NTS Bingo loosely builds on the original Bingo idea, where participants get cards with numbers printed onto them. There are a variety of different cards and not all possible numbers are contained on each card. A game leader uses some kind of random mechanism to draw numbers from a pool of possible numbers. Players mark the drawn number, if it is on their own card(s). If a player can mark a full vertical, horizontal or vertical line on his or her card, the player calls “Bingo” and wins a prize, when she or he is the first to do so in the round.

In NTS Bingo, the numbers on a card are replaced by ANTSdk elements. The drawing of numbers and their announcement is replaced by showing video clips to participants, who “mark” an element by writing down observations from the video they are shown. Thus, there is a key difference to real Bingo: Players of NTS Bingo need to identify their “hits” by themselves (subjectively)—using the mental concepts they formed based on the ANTSdk elements to categorizing entities, typically actions, into ANTSdk elements. The aim of NTS Bingo is for the players to fill in their NTS Bingo card as quickly as they can with behavioural examples of people they see in a video clip. Entities that can be categorized typically address not only the interaction between people but also how people interact with devices, machines, or other elements of the environment.

NTS Bingo is suitable for groups of four to eight players and a game leader. It is possible to have several parallel groups in settings with a larger number of participants, but still all watching the same video clip. One member of each group is the game leader for each round and this task can rotate. It is desirable, however, that the game leader is experienced in NTS. The game leader should be able to point out potential misconceptions and over- and undergeneralizations the players might hold, and thus would need some NTS expertise.

Each player gets a NTS Bingo card for each round. A set of NTS Bingo cards can be found in Additional file 1 for this paper. Table 1 provides instructions for how to create a new set of cards if other NTS frameworks are to be used during the NTS Bingo. Each card has five ANTSdk elements printed on it with some space to write notes. The same element can be printed onto one card up to three times. A random process was used to create the 63 different cards. There are empty cards that can be used to create new combinations of elements on a card.

**Table 1:** Creating your own set of NTS Bingo cards

1) Decide how many different cards you want to create, for example 50. 2) Decide how many elements should be on one card, for example 5. 3) Specify how many elements you want to distribute across the cards, for example 15 ANTSdk elements [4]. 4) Use a spread sheet programme and create the sequence from 1 to 15, with 15 rows within one column. Copy this sequence at the bottom of the column so that you end up with 250 rows that repeatedly contain the sequence from 1 to 15. Copy all used cells in this column. 5) Open a “list randomizer”, for example https://www.random.org/lists/. Paste your figures into the field offered for input. Click “randomize” and copy the ready list from the website. 6) Paste the randomized list into your spread sheet programme. 7) Use the “Search and Replace” function to change the numbers in your file against the elements that you want to have on your cards, for example replace “6” with “Gathering information”. Replace all the figures with the respective text strings. Note that you might have to check “entire cell only” or similar as search condition, to avoid replacing the “6” in “16”. You end up with a list that has 250 rows with a random sequence of the elements you want to have on your cards. 8) Creating empty lines for the notes: Create a new “sorting” column next to your “elements column”. The sorting column should contain the running figures from 1 to 250. Copy all the 250 rows one more time below, within the same sorting column. Click in the first cell of your “sorting” column and sort your file according to this column, ascending. This will create automatically an empty line after each of your elements in your “elements column”. 9) Open you word processor and create a table with 10 rows and 1 column. Refine the layout, if desired, and provide the instructions. Copy this table 50 times. Copy the first five elements from your spread sheet “elements row” and paste them into the first empty table in your word processor. Copy the next five elements into the next card and so on. Repeat until all word processor tables are filled.	

The game leader or a helper starts the video to be observed. Table 2 provides a list of video clips that can be used. While watching the video, the players try to find entities in the video that are examples for each of the ANTSdk elements on their card. This is typically a good or poor behaviour of how the interaction described in the ANTSdk element was implemented in the video. It could also be a description of why an ANTSdk element was missing in the video and how the actions would have taken a different course in response to those respective actions. The players are required to describe in detail, why they think a certain entity in the video is an example for the respective ANTSdk element. A good rule of thumb is to write the note in a way that the player could use later in a feedback conversation if that player had an opportunity to talk to the people seen in the video. This will help in explaining one’s categorization in the “defence”.

**Table 2:** A collection of possible video clips to be used in NTS Bingo

Categories	Elements	Examples for suitable video clips	Examples of observations that can be used to illustrate an ANTSdk element
Situation awareness	Gathering information	Showing difficulties in collecting all the necessary information, because of information overload or an unfavourable ratio of signal to noise. Showing different information channels that can be used and valued differently—for example, relying more on vision than on sound.	Master and Commander: Showing the shadow of the enemy ship in the fog, only a simple telescope to collect the information. Braveheart: William Wallace collects information about the emotions of the Scots to proceed in the best possible way. The second largest vessel: The team gets information about an object in the way of their ship.
	Recognizing and understanding contexts	Showing that very similar data (sounds) might mean very different things (information) in different contexts. Misunderstandings between people based on the different understanding of the same information.	The new ambulance: The “same” sound from the defibrillator and the parking support system needs to be interpreted very differently. The second largest vessel: Interpret a radar signal in very different ways, based on an overall understanding of the situation.
	Anticipating and thinking ahead	Any clip that has a surprise in it, any development that does not follow the convention. Clips that play with the stereotypes, we have as humans.	Master and Commander: The role of intuition and gut feeling, shortly before the first canon is fired. Magic Disappearance Trick: A very drastic example of what can happen from out of the blue, if there is too much inattention.
	Demonstrating self-awareness	Clips that depict some kind of self insight or the lack of it.	Scenes from “Inside Out” depicting inner dialogue that one might have while acting.
Decision making	Identifying options	Clips that depict the challenge of choosing between many options, options that have advantages and disadvantages at the same time or showing the “cost” of deciding in terms of the risk that is necessary to achieve goals.	The new ambulance: Not identifying the correct options when hearing the beeping sound with the second patient. Braveheart: William Wallace explains the challenges for the Scots to fight the English in terms of short-term and long-term losses and gains. The second largest vessel: The captain spells out the option of what will happen if the other “ship” is not moving.
	Choosing, communicating and implementing decisions	Scenes that depict how decisions are communicated to the others in the team. Convincing others to support a decision.	Master and Commander: The ship’s crew put pressure on the two young officers to communicate a clear decision—impressively so, by using only non-verbal cues.
	Reassessing decisions	Discussions of decisions taken. Review of the anticipated results in the light of the real results.	Landing John Wayne Style: There is little doubt in this scene that one of the characters disagrees with a decision.
Team work	Exchanging information	Clips that show difficulties in exchanging the relevant information between persons involved. Clips that address challenges in interacting with a device. Clips, where only a subset of those involved possess the necessary information and do not share it with all involved.	The second largest vessel: It is interesting to analyse the answer by the lighthouse keeper, merely reacting to the sentences of the captain, without exchanging the most important part of the information, namely that he is in the not-moveable lighthouse. Captain presenting himself clearly stating competence. The lighthouse keeper does not help in getting a common understanding of the situation. “Inside Out” shows different mind-sets, different ways of seeing and verbalizing the same situation. The real issue is newer discussed because of automatic reactions.
	Assessing competencies	Clips that show a direct or indirect assessment of others and what kind of implications the outcome of the assessment has.	Master and Commander: The crew quietly judges the two young officers discussing how to proceed.
	Coordinating activities	Clips that show how persons relate their activities to each other.	The second largest vessel: All involved need to find out, how they can avoid a collision.
	Supporting others	Verbal and non-verbal support of people in the group.	The Shirtless Dancing Guy: The lone nut is supported to be a leader by the first follower.
Leadership	Planning and preparing	Scenes that depict the planning of an event and what can be done in case the plan fails.	Apollo 13: “Flight” leads the discussion of how to get the damaged space capsule home again. The second largest vessel: The team should have known there would be a light house in the way.
	Prioritizing	Clips showing conflicting options or options that cannot be implemented at the same time, because of limited resources.	Apollo 13: The team on the ground discusses which electrical system should be prioritized to get the remaining power in the capsule. The Shirtless Dancing Guy: Some people prioritize the safety of the crowd and to not engage in the role of “first follower”.
	Identifying and utilizing resources	Scenes in which people or equipment is used in a useful, maybe unconventional way.	Apollo 13: The team on the ground looks at the material available in the capsule in a new light, identifying how t they can best use these resources that they already have…
	Using authority and assertiveness	Scenes that show leaders setting the direction of action. Scenes that question the decision of others/superior to them or point out an aspect overlooked so far.	Landing John Wayne Style: It might be over the top, but shows the use of authority. The second largest vessel: Provides a good example, where the personal authority is just not good enough, when meeting with the real world.
	Providing and maintaining standards	Clips that show a great skill in doing a task, but where it is questionable, whether the task should have been performed in this way at all.	The new ambulance: The ambulance gets so skilled in what they do that they fail to challenge their automatic responses.
• Master and Commander [38], use first 5 min of the movie • The second largest vessel [39], search for “lighthouse vs. ship” or “second largest vessel” • The Magic Disappearance [40]*, search for “making magic in traffic with a smartphone” • Braveheart [41], search for “Braveheart freedom speech” • Landing John Wayne Style [42], search for “How John Wayne would have handled the Jet Blue pilot” • Inside Out [43], search for “inside out dinner scene” • Apollo 13 [44], search for “Apollo 13, build a filter” or “Apollo 13, Square Peg in a Round Hole” • The new ambulance [45], search for “Funny ambulance commercial”			

ANTSdk categories and ANTSdk elements are taken from Jepsen and colleagues [6]. In the third column, we describe possible clips in overarching terms to help in finding further examples. For the examples in the fourth column, we assume that readers have already seen the video clips

Consider a fictitious example. You see a woman driving a car on a freeway. She is approaching an exit. She almost passed it, and then she looks surprised. She checks the mirror shortly, brakes hard, turns with screeching tires and just barely makes the exit.

Recognizing the exit could be noted under “gathering information”. The same would go for checking the mirror.

The surprised look on her face might be an indicator for a lack of “planning ahead” or a lack of “prioritizing”, making inferences from observable behaviour to inner states.

The first player to have an example for all ANTSdk elements on his or her card calls “Bingo”. This often happens only after the video has ended. The game leader keeps track of the sequence in which the players call “Bingo”, if more than one call of “Bingo” is made. It can be beneficial to give the group a bit of time after the video is finished until all either have a Bingo or until they indicate that they have given up or that they have not found any more examples in the video clip.

The game leader then asks the person who first called the “Bingo” to “defend” their examples written on their card. The aim of this “defence” is to stimulate a discussion of how the video relates to the elements on the card. It is good to involve all players in the discussion. The defence is meant to stimulate discussion, is highly subjective, and ideally handled in a positive, yet constructively challenging, atmosphere. The game leader and other players should request convincing explanations as to why the caller thinks that her or his observation could be seen as an example for an ANTSdk element. The game leader needs to balance an early acceptance of a defence in order to keep the flow in the game and in order to preserve a positive atmosphere on the one hand with requesting a more elaborate discussion of each example on the other hand. Early acceptance of the defence might be related to positive emotions for the defender and yet might prevent deeper analysis. On a theoretical basis, the correct classification of an entity as non-example of an ANTSdk element is an important step in concept learning. The more arguments are exchanged to classify an entity as an example or non-example of an ANTSdk element, the better should the concept learning be. On the other hand, participants might also lose interest, when too many details are requested. We count on the skills of the game leader to adjust the guidance of the discussion here.

The game leader can assist with some questions (see Table 3 for ideas) to keep the discussion going. The game leaders then decide whether they accept the “defence” of the solution by the person who first called “Bingo” or whether the next caller gets the chance for the defence and so on until a convincing solution is found or all the players have explained their thoughts. The first convincing defence wins a small prize. If no defence is convincing, then no player gets the prize and the next round is played. It is also possible to consider other defences after the first one was accepted.

**Table 3:** Examples for questions to stimulate the discussion during a NTS Bingo “defence”

• Where did you see positive and negative examples for the ANTSdk element that we are discussing? • What could be non-examples for the ANTSdk element? • Did anybody else see a different example? • Do all agree about the assessment of the example as a good or bad one? • What should have happened in the movie so that you would have put this example on your card as a good example, not a bad one (or vice versa)? • On a scale from 0 (very bad) to 10 (very good)—how good do you think this example was in regard to the safety in this situation? What should happen to improve the example by 2 points on your scale? What other criteria could you use to assess good or bad in this situation? • How did the example that we discuss contribute to the overall progress and outcome of the scene that we saw? • How is the example that we discuss related to the other examples on your own card and/or the other players’ cards? • Please describe where you had similar experiences as we saw in the example.	

## Timing

Typically, the discussion of the first video clip will take about 8–10 min. The tendency is for the discussions to go faster on further rounds. Table 4 provides a sample timing for a NTS Bingo workshop. In our experience, it is a good idea to have about four to five rounds of Bingo, with a different video clip in each round. The more the clips differ, the more chances the players get to explore the ANTSdk elements in different situations, which should support the generalization of the concepts they form [34]. This is because they are applying their concepts in different contexts. It would likely be helpful if participants are somewhat familiar with the ANTSdk elements [6] (or any other framework that is used during the Bingo) before they play NTS Bingo, as the first step in concept learning is a basic understanding of definitions. Participants could for example be asked to read a paper describing the principles before attending the workshop.

**Table 4:** Overview of a NTS Bingo workshop of 75-min duration

05 min	Set the scene for the workshop with introduction of the faculty and explaining the basic idea of the conduct
10 min	Review of the ANTSdk framework
10 min	Common example—show a video clip and discuss in plenary, what participants saw in the clip. Model how you would react to the “defence” of the example. Supplement the discussion with own examples and non-examples. Clarify questions
10 min	First Bingo round: seeing the video and doing the “defence”
09 min	Second Bingo round: seeing the video and doing the “defence”
09 min	Third Bingo round: seeing the video and doing the “defence”
08 min	Fourth Bingo round: seeing the video and doing the “defence”
05 min	Fifth Bingo round: seeing the video and doing the “defence”
09 min	Concluding discussion

## Legal considerations

The use of videos potentially relates to the legal issues of using copyrighted material without getting permission to do so and without paying licence fees. Ideally, permissions are sought and licence fees are paid. In some cases, this is not possible, for example, in those cases where the creator of a video clip is not identifiable. Here the rules of “fair use” are relevant. As our main source of video clips is YouTube, we refer to their four principles published on “fair use” [35]:

1. The purpose of using the material should be educational and not for commercial purposes.
2. The nature of the work—where factual clips are less likely to violate the fair use principle than fictional work.
3. The proportion of the material used: smaller parts are less likely to violate fair use—especially if the piece shown is not the “heart” of the complete work.
4. The effect the use has on potential financial interests of the copyright owner.

Based on these principles, we consider the use of video clips in this workshop as fair, because:

1. The clips are used with the educational purpose of helping healthcare professionals to learn NTS concepts and so better help patients.
2. While the video clips suggested are partly fictional, some of them have been created to promote commercial products.
3. We only use small clips from larger movies, mostly not the “heart”.
4. The short clips we use are not intended to be sold. Many of the video clips were made to be distributed widely. Showing them would in the best case support the intent for which they were made. Showing clips of larger movies might actually trigger workshop participants to buy and watch the whole movie.

## Suitable video clips

Above we discussed in general why NTS Bingo might help in concept learning. Here we discuss the content side of the video clips. It might be beneficial to remove the healthcare contents in some learning sessions on NTS, because by removing the healthcare content, it appears to be easier to concentrate on the NTS aspects. Out-of-context discussions can reduce potential overload from a mix of clinical considerations and trying to grasp new concepts at the same time [36]. The discussion of NTS in an out-of-context situation may increase psychological safety because misconceptions can be clarified in a situation, where the professional self-image is not at stake. Participants from various backgrounds can participate on “equal grounds”. The video clips that we suggest using in NTS Bingo often use caricature or drama elements and thus make certain points more clearly than documentary clips. Alienation, seeing an interaction in different contexts, might facilitate concept learning and applicability in real healthcare situations, because the relevant features of a concept become clearer by using it in such diverse categorization tasks [37]. On the other side, such exercises require to help participants make a “far transfer” [34] from the learning situation to their clinical work: participants will have to adapt what they learned during the game to their clinical work.

In many of the videos that work well with NTS Bingo, one or more persons are depicted in situations that most of us would not necessarily share with others. In most of the videos we use, this is staged and nobody gets hurt or embarrassed for real. However, some videos also depict real-world challenges and we strive to find a balance between having a video that is usable to make a strong point and an ethical treatment of videos of others. The legal and cultural perspective in the selection of the video clips needs to be taken into account, depending on the country in which NTS Bingo is played. What might count as a funny example in one context might violate cultural norms in a different context.

The definition of the categories and elements in ANTSdk and similar systems are not mutually exclusive, and the elements are related to each other. Consequently, many video clips will be relevant for several elements and one element can be illustrated with different video clips. Theoretically speaking, this suggests that NTS Bingo is better suited for learners with an already existing basic understanding of NTS, who are able to do basic categorizations of entities as examples or non-examples of ANTSdk elements. Without such a basic understanding, it might be difficult to distinguish examples from non-examples of an ANTSdk element.

Ambiguous video clips are very interesting—those that could only be interpreted after knowing the motives of the acting persons. This is often the case in clips with little context information and where the persons involved do not talk about their motives but mainly act. Such clips can stimulate elaborate reflections regarding their interpretation.

## Discussion

### Potential risks and limitations

NTS Bingo is intended to help learners acquire the NTS concepts so that they can apply them in their clinical and teaching practice. A potential risk is that participants build misconceptions or over- or undergeneralise. For this reason, it is beneficial that a game leader is aware of those risks and intervenes in a corrective manner, if needed. On the other hand, the ANTSdk elements are self-explaining to some extent and relate to common sense so that we assume this risk not to be very high. By presenting and discussing different viewpoints in the defence, we assume that potential errors can be caught.

The selection of the video clips is culturally sensitive and we thus suggest to consider which clips can be shown in which context. We described the legal side and related challenges above.

## Lessons learned from the conduct of NTS Bingo

As mentioned earlier, we did not evaluate NTS Bingo formally. Here we describe some take home messages that we drew from our experience of running NTS Bingo sessions:

For some participants, it takes one or two rounds to get familiar with the procedures of NTS Bingo. We therefore systematically include a trial example, where we demonstrate the procedures in the session introduction in a step-by-step fashion.

Some participants do not immediately see the point of using non-medical video clips. On the other hand, we also received feedback that this particular aspect was valuable for participants—especially in combination with very different clips that allow participants to apply the NTS concepts in different contexts.

A potential challenge is the need to balance the conceptual precision around the NTS concepts while keeping a flow in the game during the defence. As described above, we would accept some degree of misconception, under- and overgeneralizations of the NTS concepts in order to stimulate participants’ reflections and in order to keep the flow in the discussion. We recommend that someone with NTS expertise who is also familiar with facilitating group discussions be assigned the role of game leader to ensure the success of the session.

Enjoy playing NTS Bingo.

## Abbreviations

NTS, non-technical skills; ANTSdk, Anaesthesia Non-Technical Skills System – Danish Version; CRM, crisis resource management

## Additional file

Additional file 1: Non-Technical Skills Bingo Cards. (DOCX 96 kb)
