# Supplementary material for: Non-Technical Skills Bingo—a game to facilitate the learning of complex concepts
Source: Adv Simul (Lond). 2016 Jul 22;1:23. doi: 10.1186/s41077-016-0024-z (PMC5806249; doi:10.1186/s41077-016-0024-z)
Supplement: Supplementary file 1 — Non-Technical Skills Bingo Cards. (DOCX 96 kb) [file 41077_2016_24_MOESM1_ESM.docx]

Non-Technical Skills Bingo

Dieckmann et al, Advances in Simulation (2016). DOI: 10.1186/s41077-016-0024-z

|  | **Card 1** |
| --- | --- |
| 1 | Gathering information |
|  |  |
| 2 | Recognising and understanding contexts |
|  |  |
| 3 | Providing and maintaining standards |
|  |  |
| 4 | Recognising and understanding contexts |
|  |  |
| 5 | Anticipating and thinking ahead |
|  |  |

https://www.regionh.dk/CAMES/Forskning/Forskningsprojekter/Sider/ANTSdk.aspx

# Instructions

Use this card for the movie scene that you will see. Take notes in the NTS element for which you saw a good or a bad behavioural example. Such behaviour can also be in relation to how the people acting in the scene interact with devices or other parts of the environment. When you have completed all the boxes with examples shout ‘Bingo’. You will then be asked to describe the behaviours associated with each NTS element. If you have the same element listed more than once then you will have to provide a separate example for each.

Non-Technical Skills Bingo

Dieckmann et al, Advances in Simulation (2016). DOI: 10.1186/s41077-016-0024-z

|  | Card 2 |
| --- | --- |
| 1 | Anticipating and thinking ahead |
|  |  |
| 2 | Anticipating and thinking ahead |
|  |  |
| 3 | Demonstrating self-awareness |
|  |  |
| 4 | Identifying options |
|  |  |
| 5 | Choosing, communicating and implementing decisions |
|  |  |

https://www.regionh.dk/CAMES/Forskning/Forskningsprojekter/Sider/ANTSdk.aspx

# Instructions

Use this card for the movie scene that you will see. Take notes in the NTS element for which you saw a good or a bad behavioural example. Such behaviour can also be in relation to how the people acting in the scene interact with devices or other parts of The environment. When you have completed all the boxes with examples shout ‘Bingo’. You will then be asked to describe the behaviours associated with each NTS element. If you have the same element listed more than once then you will have to provide a separate example for each.

Non-Technical Skills Bingo

Dieckmann et al, Advances in Simulation (2016). DOI: 10.1186/s41077-016-0024-z

|  | **Card 3** |
| --- | --- |
| 1 | Exchanging information |
|  |  |
| 2 | Reassessing decisions |
|  |  |
| 3 | Gathering information |
|  |  |
| 4 | Assessing Competencies |
|  |  |
| 5 | Coordinating activities |
|  |  |

https://www.regionh.dk/CAMES/Forskning/Forskningsprojekter/Sider/ANTSdk.aspx

# Instructions

Use this card for the movie scene that you will see. Take notes in the NTS element for which you saw a good or a bad behavioural example. Such behaviour can also be in relation to how the people acting in the scene interact with devices or other parts of The environment. When you have completed all the boxes with examples shout ‘Bingo’. You will then be asked to describe the behaviours associated with each NTS element. If you have the same element listed more than once then you will have to provide a separate example for each.

Non-Technical Skills Bingo

Dieckmann et al, Advances in Simulation (2016). DOI: 10.1186/s41077-016-0024-z

|  | Card 4 |
| --- | --- |
| 1 | Identifying options |
|  |  |
| 2 | Exchanging information |
|  |  |
| 3 | Assessing Competencies |
|  |  |
| 4 | Supporting others |
|  |  |
| 5 | Recognising and understanding contexts |
|  |  |

https://www.regionh.dk/CAMES/Forskning/Forskningsprojekter/Sider/ANTSdk.aspx

# Instructions

Use this card for the movie scene that you will see. Take notes in the NTS element for which you saw a good or a bad behavioural example. Such behaviour can also be in relation to how the people acting in the scene interact with devices or other parts of The environment. When you have completed all the boxes with examples shout ‘Bingo’. You will then be asked to describe the behaviours associated with each NTS element. If you have the same element listed more than once then you will have to provide a separate example for each.

Non-Technical Skills Bingo

Dieckmann et al, Advances in Simulation (2016). DOI: 10.1186/s41077-016-0024-z

|  | **Card 5** |
| --- | --- |
| 1 | Reassessing decisions |
|  |  |
| 2 | Recognising and understanding contexts |
|  |  |
| 3 | Prioritizing |
|  |  |
| 4 | Demonstrating self-awareness |
|  |  |
| 5 | Planning and preparing |
|  |  |

https://www.regionh.dk/CAMES/Forskning/Forskningsprojekter/Sider/ANTSdk.aspx

# Instructions

Use this card for the movie scene that you will see. Take notes in the NTS element for which you saw a good or a bad behavioural example. Such behaviour can also be in relation to how the people acting in the scene interact with devices or other parts of The environment. When you have completed all the boxes with examples shout ‘Bingo’. You will then be asked to describe the behaviours associated with each NTS element. If you have the same element listed more than once then you will have to provide a separate example for each.

Non-Technical Skills Bingo

Dieckmann et al, Advances in Simulation (2016). DOI: 10.1186/s41077-016-0024-z

|  | Card 6 |
| --- | --- |
| 1 | Coordinating activities |
|  |  |
| 2 | Choosing, communicating and implementing decisions |
|  |  |
| 3 | Demonstrating self-awareness |
|  |  |
| 4 | Exchanging information |
|  |  |
| 5 | Gathering information |
|  |  |

https://www.regionh.dk/CAMES/Forskning/Forskningsprojekter/Sider/ANTSdk.aspx

# Instructions

Use this card for the movie scene that you will see. Take notes in the NTS element for which you saw a good or a bad behavioural example. Such behaviour can also be in relation to how the people acting in the scene interact with devices or other parts of The environment. When you have completed all the boxes with examples shout ‘Bingo’. You will then be asked to describe the behaviours associated with each NTS element. If you have the same element listed more than once then you will have to provide a separate example for each.

Non-Technical Skills Bingo

Dieckmann et al, Advances in Simulation (2016). DOI: 10.1186/s41077-016-0024-z

|  | **Card 7** |
| --- | --- |
| 1 | Choosing, communicating and implementing decisions |
|  |  |
| 2 | Exchanging information |
|  |  |
| 3 | Recognising and understanding contexts |
|  |  |
| 4 | Providing and maintaining standards |
|  |  |
| 5 | Prioritizing |
|  |  |

https://www.regionh.dk/CAMES/Forskning/Forskningsprojekter/Sider/ANTSdk.aspx

# Instructions

Use this card for the movie scene that you will see. Take notes in the NTS element for which you saw a good or a bad behavioural example. Such behaviour can also be in relation to how the people acting in the scene interact with devices or other parts of The environment. When you have completed all the boxes with examples shout ‘Bingo’. You will then be asked to describe the behaviours associated with each NTS element. If you have the same element listed more than once then you will have to provide a separate example for each.

Non-Technical Skills Bingo

Dieckmann et al, Advances in Simulation (2016). DOI: 10.1186/s41077-016-0024-z

|  | Card 8 |
| --- | --- |
| 1 | Supporting others |
|  |  |
| 2 | Identifying options |
|  |  |
| 3 | Choosing, communicating and implementing decisions |
|  |  |
| 4 | Gathering information |
|  |  |
| 5 | Supporting others |
|  |  |

https://www.regionh.dk/CAMES/Forskning/Forskningsprojekter/Sider/ANTSdk.aspx

# Instructions

Use this card for the movie scene that you will see. Take notes in the NTS element for which you saw a good or a bad behavioural example. Such behaviour can also be in relation to how the people acting in the scene interact with devices or other parts of The environment. When you have completed all the boxes with examples shout ‘Bingo’. You will then be asked to describe the behaviours associated with each NTS element. If you have the same element listed more than once then you will have to provide a separate example for each.

Non-Technical Skills Bingo

Dieckmann et al, Advances in Simulation (2016). DOI: 10.1186/s41077-016-0024-z

|  | **Card 9** |
| --- | --- |
| 1 | Exchanging information |
|  |  |
| 2 | Identifying and utilizing resources |
|  |  |
| 3 | Supporting others |
|  |  |
| 4 | Assessing Competencies |
|  |  |
| 5 | Choosing, communicating and implementing decisions |
|  |  |

https://www.regionh.dk/CAMES/Forskning/Forskningsprojekter/Sider/ANTSdk.aspx

# Instructions

Use this card for the movie scene that you will see. Take notes in the NTS element for which you saw a good or a bad behavioural example. Such behaviour can also be in relation to how the people acting in the scene interact with devices or other parts of The environment. When you have completed all the boxes with examples shout ‘Bingo’. You will then be asked to describe the behaviours associated with each NTS element. If you have the same element listed more than once then you will have to provide a separate example for each.

Non-Technical Skills Bingo

Dieckmann et al, Advances in Simulation (2016). DOI: 10.1186/s41077-016-0024-z

|  | Card 10 |
| --- | --- |
| 1 | Assessing Competencies |
|  |  |
| 2 | Choosing, communicating and implementing decisions |
|  |  |
| 3 | Planning and preparing |
|  |  |
| 4 | Exchanging information |
|  |  |
| 5 | Demonstrating self-awareness |
|  |  |

https://www.regionh.dk/CAMES/Forskning/Forskningsprojekter/Sider/ANTSdk.aspx

# Instructions

Use this card for the movie scene that you will see. Take notes in the NTS element for which you saw a good or a bad behavioural example. Such behaviour can also be in relation to how the people acting in the scene interact with devices or other parts of The environment. When you have completed all the boxes with examples shout ‘Bingo’. You will then be asked to describe the behaviours associated with each NTS element. If you have the same element listed more than once then you will have to provide a separate example for each.

Non-Technical Skills Bingo

Dieckmann et al, Advances in Simulation (2016). DOI: 10.1186/s41077-016-0024-z

|  | **Card 11** |
| --- | --- |
| 1 | Planning and preparing |
|  |  |
| 2 | Coordinating activities |
|  |  |
| 3 | Supporting others |
|  |  |
| 4 | Supporting others |
|  |  |
| 5 | Identifying and utilizing resources |
|  |  |

https://www.regionh.dk/CAMES/Forskning/Forskningsprojekter/Sider/ANTSdk.aspx

# Instructions

Use this card for the movie scene that you will see. Take notes in the NTS element for which you saw a good or a bad behavioural example. Such behaviour can also be in relation to how the people acting in the scene interact with devices or other parts of The environment. When you have completed all the boxes with examples shout ‘Bingo’. You will then be asked to describe the behaviours associated with each NTS element. If you have the same element listed more than once then you will have to provide a separate example for each.

Non-Technical Skills Bingo

Dieckmann et al, Advances in Simulation (2016). DOI: 10.1186/s41077-016-0024-z

|  | Card 12 |
| --- | --- |
| 1 | Demonstrating self-awareness |
|  |  |
| 2 | Gathering information |
|  |  |
| 3 | Exchanging information |
|  |  |
| 4 | Gathering information |
|  |  |
| 5 | Reassessing decisions |
|  |  |

https://www.regionh.dk/CAMES/Forskning/Forskningsprojekter/Sider/ANTSdk.aspx

# Instructions

Use this card for the movie scene that you will see. Take notes in the NTS element for which you saw a good or a bad behavioural example. Such behaviour can also be in relation to how the people acting in the scene interact with devices or other parts of The environment. When you have completed all the boxes with examples shout ‘Bingo’. You will then be asked to describe the behaviours associated with each NTS element. If you have the same element listed more than once then you will have to provide a separate example for each.

Non-Technical Skills Bingo

Dieckmann et al, Advances in Simulation (2016). DOI: 10.1186/s41077-016-0024-z

|  | **Card 13** |
| --- | --- |
| 1 | Prioritizing |
|  |  |
| 2 | Prioritizing |
|  |  |
| 3 | Assessing Competencies |
|  |  |
| 4 | Prioritizing |
|  |  |
| 5 | Planning and preparing |
|  |  |

https://www.regionh.dk/CAMES/Forskning/Forskningsprojekter/Sider/ANTSdk.aspx

# Instructions

Use this card for the movie scene that you will see. Take notes in the NTS element for which you saw a good or a bad behavioural example. Such behaviour can also be in relation to how the people acting in the scene interact with devices or other parts of The environment. When you have completed all the boxes with examples shout ‘Bingo’. You will then be asked to describe the behaviours associated with each NTS element. If you have the same element listed more than once then you will have to provide a separate example for each.

Non-Technical Skills Bingo

Dieckmann et al, Advances in Simulation (2016). DOI: 10.1186/s41077-016-0024-z

|  | Card 14 |
| --- | --- |
| 1 | Assessing Competencies |
|  |  |
| 2 | Supporting others |
|  |  |
| 3 | Exchanging information |
|  |  |
| 4 | Planning and preparing |
|  |  |
| 5 | Assessing Competencies |
|  |  |

https://www.regionh.dk/CAMES/Forskning/Forskningsprojekter/Sider/ANTSdk.aspx

# Instructions

Use this card for the movie scene that you will see. Take notes in the NTS element for which you saw a good or a bad behavioural example. Such behaviour can also be in relation to how the people acting in the scene interact with devices or other parts of The environment. When you have completed all the boxes with examples shout ‘Bingo’. You will then be asked to describe the behaviours associated with each NTS element. If you have the same element listed more than once then you will have to provide a separate example for each.

Non-Technical Skills Bingo

Dieckmann et al, Advances in Simulation (2016). DOI: 10.1186/s41077-016-0024-z

|  | **Card 15** |
| --- | --- |
| 1 | Demonstrating self-awareness |
|  |  |
| 2 | Identifying and utilizing resources |
|  |  |
| 3 | Prioritizing |
|  |  |
| 4 | Reassessing decisions |
|  |  |
| 5 | Recognising and understanding contexts |
|  |  |

https://www.regionh.dk/CAMES/Forskning/Forskningsprojekter/Sider/ANTSdk.aspx

# Instructions

Use this card for the movie scene that you will see. Take notes in the NTS element for which you saw a good or a bad behavioural example. Such behaviour can also be in relation to how the people acting in the scene interact with devices or other parts of The environment. When you have completed all the boxes with examples shout ‘Bingo’. You will then be asked to describe the behaviours associated with each NTS element. If you have the same element listed more than once then you will have to provide a separate example for each.

Non-Technical Skills Bingo

Dieckmann et al, Advances in Simulation (2016). DOI: 10.1186/s41077-016-0024-z

|  | Card 16 |
| --- | --- |
| 1 | Exchanging information |
|  |  |
| 2 | Recognising and understanding contexts |
|  |  |
| 3 | Recognising and understanding contexts |
|  |  |
| 4 | Reassessing decisions |
|  |  |
| 5 | Identifying options |
|  |  |

https://www.regionh.dk/CAMES/Forskning/Forskningsprojekter/Sider/ANTSdk.aspx

# Instructions

Use this card for the movie scene that you will see. Take notes in the NTS element for which you saw a good or a bad behavioural example. Such behaviour can also be in relation to how the people acting in the scene interact with devices or other parts of The environment. When you have completed all the boxes with examples shout ‘Bingo’. You will then be asked to describe the behaviours associated with each NTS element. If you have the same element listed more than once then you will have to provide a separate example for each.

Non-Technical Skills Bingo

Dieckmann et al, Advances in Simulation (2016). DOI: 10.1186/s41077-016-0024-z

|  | **Card 17** |
| --- | --- |
| 1 | Prioritizing |
|  |  |
| 2 | Supporting others |
|  |  |
| 3 | Exchanging information |
|  |  |
| 4 | Gathering information |
|  |  |
| 5 | Coordinating activities |
|  |  |

https://www.regionh.dk/CAMES/Forskning/Forskningsprojekter/Sider/ANTSdk.aspx

# Instructions

Use this card for the movie scene that you will see. Take notes in the NTS element for which you saw a good or a bad behavioural example. Such behaviour can also be in relation to how the people acting in the scene interact with devices or other parts of The environment. When you have completed all the boxes with examples shout ‘Bingo’. You will then be asked to describe the behaviours associated with each NTS element. If you have the same element listed more than once then you will have to provide a separate example for each.

Non-Technical Skills Bingo

Dieckmann et al, Advances in Simulation (2016). DOI: 10.1186/s41077-016-0024-z

|  | Card 18 |
| --- | --- |
| 1 | Choosing, communicating and implementing decisions |
|  |  |
| 2 | Anticipating and thinking ahead |
|  |  |
| 3 | Gathering information |
|  |  |
| 4 | Choosing, communicating and implementing decisions |
|  |  |
| 5 | Identifying and utilizing resources |
|  |  |

https://www.regionh.dk/CAMES/Forskning/Forskningsprojekter/Sider/ANTSdk.aspx

# Instructions

Use this card for the movie scene that you will see. Take notes in the NTS element for which you saw a good or a bad behavioural example. Such behaviour can also be in relation to how the people acting in the scene interact with devices or other parts of The environment. When you have completed all the boxes with examples shout ‘Bingo’. You will then be asked to describe the behaviours associated with each NTS element. If you have the same element listed more than once then you will have to provide a separate example for each.

Non-Technical Skills Bingo

Dieckmann et al, Advances in Simulation (2016). DOI: 10.1186/s41077-016-0024-z

|  | **Card 19** |
| --- | --- |
| 1 | Planning and preparing |
|  |  |
| 2 | Anticipating and thinking ahead |
|  |  |
| 3 | Demonstrating self-awareness |
|  |  |
| 4 | Coordinating activities |
|  |  |
| 5 | Prioritizing |
|  |  |

https://www.regionh.dk/CAMES/Forskning/Forskningsprojekter/Sider/ANTSdk.aspx

# Instructions

Use this card for the movie scene that you will see. Take notes in the NTS element for which you saw a good or a bad behavioural example. Such behaviour can also be in relation to how the people acting in the scene interact with devices or other parts of The environment. When you have completed all the boxes with examples shout ‘Bingo’. You will then be asked to describe the behaviours associated with each NTS element. If you have the same element listed more than once then you will have to provide a separate example for each.

Non-Technical Skills Bingo

Dieckmann et al, Advances in Simulation (2016). DOI: 10.1186/s41077-016-0024-z

|  | Card 20 |
| --- | --- |
| 1 | Choosing, communicating and implementing decisions |
|  |  |
| 2 | Supporting others |
|  |  |
| 3 | Identifying and utilizing resources |
|  |  |
| 4 | Coordinating activities |
|  |  |
| 5 | Choosing, communicating and implementing decisions |
|  |  |

https://www.regionh.dk/CAMES/Forskning/Forskningsprojekter/Sider/ANTSdk.aspx

# Instructions

Use this card for the movie scene that you will see. Take notes in the NTS element for which you saw a good or a bad behavioural example. Such behaviour can also be in relation to how the people acting in the scene interact with devices or other parts of The environment. When you have completed all the boxes with examples shout ‘Bingo’. You will then be asked to describe the behaviours associated with each NTS element. If you have the same element listed more than once then you will have to provide a separate example for each.

Non-Technical Skills Bingo

Dieckmann et al, Advances in Simulation (2016). DOI: 10.1186/s41077-016-0024-z

|  | **Card 21** |
| --- | --- |
| 1 | Anticipating and thinking ahead |
|  |  |
| 2 | Gathering information |
|  |  |
| 3 | Reassessing decisions |
|  |  |
| 4 | Demonstrating self-awareness |
|  |  |
| 5 | Anticipating and thinking ahead |
|  |  |

https://www.regionh.dk/CAMES/Forskning/Forskningsprojekter/Sider/ANTSdk.aspx

# Instructions

Use this card for the movie scene that you will see. Take notes in the NTS element for which you saw a good or a bad behavioural example. Such behaviour can also be in relation to how the people acting in the scene interact with devices or other parts of The environment. When you have completed all the boxes with examples shout ‘Bingo’. You will then be asked to describe the behaviours associated with each NTS element. If you have the same element listed more than once then you will have to provide a separate example for each.

Non-Technical Skills Bingo

Dieckmann et al, Advances in Simulation (2016). DOI: 10.1186/s41077-016-0024-z

|  | Card 22 |
| --- | --- |
| 1 | Demonstrating self-awareness |
|  |  |
| 2 | Coordinating activities |
|  |  |
| 3 | Using authority and assertiveness |
|  |  |
| 4 | Assessing Competencies |
|  |  |
| 5 | Gathering information |
|  |  |

https://www.regionh.dk/CAMES/Forskning/Forskningsprojekter/Sider/ANTSdk.aspx

# Instructions

Use this card for the movie scene that you will see. Take notes in the NTS element for which you saw a good or a bad behavioural example. Such behaviour can also be in relation to how the people acting in the scene interact with devices or other parts of The environment. When you have completed all the boxes with examples shout ‘Bingo’. You will then be asked to describe the behaviours associated with each NTS element. If you have the same element listed more than once then you will have to provide a separate example for each.

Non-Technical Skills Bingo

Dieckmann et al, Advances in Simulation (2016). DOI: 10.1186/s41077-016-0024-z

|  | **Card 23** |
| --- | --- |
| 1 | Identifying options |
|  |  |
| 2 | Identifying options |
|  |  |
| 3 | Providing and maintaining standards |
|  |  |
| 4 | Exchanging information |
|  |  |
| 5 | Recognising and understanding contexts |
|  |  |

https://www.regionh.dk/CAMES/Forskning/Forskningsprojekter/Sider/ANTSdk.aspx

# Instructions

Use this card for the movie scene that you will see. Take notes in the NTS element for which you saw a good or a bad behavioural example. Such behaviour can also be in relation to how the people acting in the scene interact with devices or other parts of The environment. When you have completed all the boxes with examples shout ‘Bingo’. You will then be asked to describe the behaviours associated with each NTS element. If you have the same element listed more than once then you will have to provide a separate example for each.

Non-Technical Skills Bingo

Dieckmann et al, Advances in Simulation (2016). DOI: 10.1186/s41077-016-0024-z

|  | Card 24 |
| --- | --- |
| 1 | Demonstrating self-awareness |
|  |  |
| 2 | Supporting others |
|  |  |
| 3 | Assessing Competencies |
|  |  |
| 4 | Exchanging information |
|  |  |
| 5 | Choosing, communicating and implementing decisions |
|  |  |

https://www.regionh.dk/CAMES/Forskning/Forskningsprojekter/Sider/ANTSdk.aspx

# Instructions

Use this card for the movie scene that you will see. Take notes in the NTS element for which you saw a good or a bad behavioural example. Such behaviour can also be in relation to how the people acting in the scene interact with devices or other parts of The environment. When you have completed all the boxes with examples shout ‘Bingo’. You will then be asked to describe the behaviours associated with each NTS element. If you have the same element listed more than once then you will have to provide a separate example for each.

Non-Technical Skills Bingo

Dieckmann et al, Advances in Simulation (2016). DOI: 10.1186/s41077-016-0024-z

|  | **Card 25** |
| --- | --- |
| 1 | Exchanging information |
|  |  |
| 2 | Coordinating activities |
|  |  |
| 3 | Assessing Competencies |
|  |  |
| 4 | Providing and maintaining standards |
|  |  |
| 5 | Recognising and understanding contexts |
|  |  |

https://www.regionh.dk/CAMES/Forskning/Forskningsprojekter/Sider/ANTSdk.aspx

# Instructions

Use this card for the movie scene that you will see. Take notes in the NTS element for which you saw a good or a bad behavioural example. Such behaviour can also be in relation to how the people acting in the scene interact with devices or other parts of The environment. When you have completed all the boxes with examples shout ‘Bingo’. You will then be asked to describe the behaviours associated with each NTS element. If you have the same element listed more than once then you will have to provide a separate example for each.

Non-Technical Skills Bingo

Dieckmann et al, Advances in Simulation (2016). DOI: 10.1186/s41077-016-0024-z

|  | Card 26 |
| --- | --- |
| 1 | Demonstrating self-awareness |
|  |  |
| 2 | Exchanging information |
|  |  |
| 3 | Planning and preparing |
|  |  |
| 4 | Supporting others |
|  |  |
| 5 | Choosing, communicating and implementing decisions |
|  |  |

https://www.regionh.dk/CAMES/Forskning/Forskningsprojekter/Sider/ANTSdk.aspx

# Instructions

Use this card for the movie scene that you will see. Take notes in the NTS element for which you saw a good or a bad behavioural example. Such behaviour can also be in relation to how the people acting in the scene interact with devices or other parts of The environment. When you have completed all the boxes with examples shout ‘Bingo’. You will then be asked to describe the behaviours associated with each NTS element. If you have the same element listed more than once then you will have to provide a separate example for each.

Non-Technical Skills Bingo

Dieckmann et al, Advances in Simulation (2016). DOI: 10.1186/s41077-016-0024-z

|  | **Card 27** |
| --- | --- |
| 1 | Anticipating and thinking ahead |
|  |  |
| 2 | Coordinating activities |
|  |  |
| 3 | Choosing, communicating and implementing decisions |
|  |  |
| 4 | Supporting others |
|  |  |
| 5 | Anticipating and thinking ahead |
|  |  |

https://www.regionh.dk/CAMES/Forskning/Forskningsprojekter/Sider/ANTSdk.aspx

# Instructions

Use this card for the movie scene that you will see. Take notes in the NTS element for which you saw a good or a bad behavioural example. Such behaviour can also be in relation to how the people acting in the scene interact with devices or other parts of The environment. When you have completed all the boxes with examples shout ‘Bingo’. You will then be asked to describe the behaviours associated with each NTS element. If you have the same element listed more than once then you will have to provide a separate example for each.

Non-Technical Skills Bingo

Dieckmann et al, Advances in Simulation (2016). DOI: 10.1186/s41077-016-0024-z

|  | Card 28 |
| --- | --- |
| 1 | Using authority and assertiveness |
|  |  |
| 2 | Prioritizing |
|  |  |
| 3 | Anticipating and thinking ahead |
|  |  |
| 4 | Identifying options |
|  |  |
| 5 | Identifying and utilizing resources |
|  |  |

https://www.regionh.dk/CAMES/Forskning/Forskningsprojekter/Sider/ANTSdk.aspx

# Instructions

Use this card for the movie scene that you will see. Take notes in the NTS element for which you saw a good or a bad behavioural example. Such behaviour can also be in relation to how the people acting in the scene interact with devices or other parts of The environment. When you have completed all the boxes with examples shout ‘Bingo’. You will then be asked to describe the behaviours associated with each NTS element. If you have the same element listed more than once then you will have to provide a separate example for each.

Note – behaviours can be examples of either good performance or poor performance for that principle

Non-Technical Skills Bingo

Dieckmann et al, Advances in Simulation (2016). DOI: 10.1186/s41077-016-0024-z

|  | **Card 29** |
| --- | --- |
| 1 | Supporting others |
|  |  |
| 2 | Planning and preparing |
|  |  |
| 3 | Coordinating activities |
|  |  |
| 4 | Demonstrating self-awareness |
|  |  |
| 5 | Supporting others |
|  |  |

https://www.regionh.dk/CAMES/Forskning/Forskningsprojekter/Sider/ANTSdk.aspx

# Instructions

Use this card for the movie scene that you will see. Take notes in the NTS element for which you saw a good or a bad behavioural example. Such behaviour can also be in relation to how the people acting in the scene interact with devices or other parts of The environment. When you have completed all the boxes with examples shout ‘Bingo’. You will then be asked to describe the behaviours associated with each NTS element. If you have the same element listed more than once then you will have to provide a separate example for each.

Non-Technical Skills Bingo

Dieckmann et al, Advances in Simulation (2016). DOI: 10.1186/s41077-016-0024-z

|  | Card 30 |
| --- | --- |
| 1 | Identifying options |
|  |  |
| 2 | Demonstrating self-awareness |
|  |  |
| 3 | Planning and preparing |
|  |  |
| 4 | Prioritizing |
|  |  |
| 5 | Demonstrating self-awareness |
|  |  |

https://www.regionh.dk/CAMES/Forskning/Forskningsprojekter/Sider/ANTSdk.aspx

# Instructions

Use this card for the movie scene that you will see. Take notes in the NTS element for which you saw a good or a bad behavioural example. Such behaviour can also be in relation to how the people acting in the scene interact with devices or other parts of The environment. When you have completed all the boxes with examples shout ‘Bingo’. You will then be asked to describe the behaviours associated with each NTS element. If you have the same element listed more than once then you will have to provide a separate example for each.

Non-Technical Skills Bingo

Dieckmann et al, Advances in Simulation (2016). DOI: 10.1186/s41077-016-0024-z

|  | **Card 31** |
| --- | --- |
| 1 | Planning and preparing |
|  |  |
| 2 | Prioritizing |
|  |  |
| 3 | Identifying and utilizing resources |
|  |  |
| 4 | Identifying options |
|  |  |
| 5 | Gathering information |
|  |  |

https://www.regionh.dk/CAMES/Forskning/Forskningsprojekter/Sider/ANTSdk.aspx

# Instructions

Use this card for the movie scene that you will see. Take notes in the NTS element for which you saw a good or a bad behavioural example. Such behaviour can also be in relation to how the people acting in the scene interact with devices or other parts of The environment. When you have completed all the boxes with examples shout ‘Bingo’. You will then be asked to describe the behaviours associated with each NTS element. If you have the same element listed more than once then you will have to provide a separate example for each.

Non-Technical Skills Bingo

Dieckmann et al, Advances in Simulation (2016). DOI: 10.1186/s41077-016-0024-z

|  | Card 32 |
| --- | --- |
| 1 | Anticipating and thinking ahead |
|  |  |
| 2 | Planning and preparing |
|  |  |
| 3 | Coordinating activities |
|  |  |
| 4 | Exchanging information |
|  |  |
| 5 | Reassessing decisions |
|  |  |

https://www.regionh.dk/CAMES/Forskning/Forskningsprojekter/Sider/ANTSdk.aspx

# Instructions

Use this card for the movie scene that you will see. Take notes in the NTS element for which you saw a good or a bad behavioural example. Such behaviour can also be in relation to how the people acting in the scene interact with devices or other parts of The environment. When you have completed all the boxes with examples shout ‘Bingo’. You will then be asked to describe the behaviours associated with each NTS element. If you have the same element listed more than once then you will have to provide a separate example for each.

Non-Technical Skills Bingo

Dieckmann et al, Advances in Simulation (2016). DOI: 10.1186/s41077-016-0024-z

|  | **Card 33** |
| --- | --- |
| 1 | Identifying and utilizing resources |
|  |  |
| 2 | Recognising and understanding contexts |
|  |  |
| 3 | Exchanging information |
|  |  |
| 4 | Recognising and understanding contexts |
|  |  |
| 5 | Demonstrating self-awareness |
|  |  |

https://www.regionh.dk/CAMES/Forskning/Forskningsprojekter/Sider/ANTSdk.aspx

# Instructions

Use this card for the movie scene that you will see. Take notes in the NTS element for which you saw a good or a bad behavioural example. Such behaviour can also be in relation to how the people acting in the scene interact with devices or other parts of The environment. When you have completed all the boxes with examples shout ‘Bingo’. You will then be asked to describe the behaviours associated with each NTS element. If you have the same element listed more than once then you will have to provide a separate example for each.

Non-Technical Skills Bingo

Dieckmann et al, Advances in Simulation (2016). DOI: 10.1186/s41077-016-0024-z

|  | Card 34 |
| --- | --- |
| 1 | Supporting others |
|  |  |
| 2 | Prioritizing |
|  |  |
| 3 | Using authority and assertiveness |
|  |  |
| 4 | Choosing, communicating and implementing decisions |
|  |  |
| 5 | Using authority and assertiveness |
|  |  |

https://www.regionh.dk/CAMES/Forskning/Forskningsprojekter/Sider/ANTSdk.aspx

# Instructions

Use this card for the movie scene that you will see. Take notes in the NTS element for which you saw a good or a bad behavioural example. Such behaviour can also be in relation to how the people acting in the scene interact with devices or other parts of The environment. When you have completed all the boxes with examples shout ‘Bingo’. You will then be asked to describe the behaviours associated with each NTS element. If you have the same element listed more than once then you will have to provide a separate example for each.

Non-Technical Skills Bingo

Dieckmann et al, Advances in Simulation (2016). DOI: 10.1186/s41077-016-0024-z

|  | **Card 35** |
| --- | --- |
| 1 | Coordinating activities |
|  |  |
| 2 | Using authority and assertiveness |
|  |  |
| 3 | Using authority and assertiveness |
|  |  |
| 4 | Exchanging information |
|  |  |
| 5 | Identifying options |
|  |  |

https://www.regionh.dk/CAMES/Forskning/Forskningsprojekter/Sider/ANTSdk.aspx

# Instructions

Use this card for the movie scene that you will see. Take notes in the NTS element for which you saw a good or a bad behavioural example. Such behaviour can also be in relation to how the people acting in the scene interact with devices or other parts of The environment. When you have completed all the boxes with examples shout ‘Bingo’. You will then be asked to describe the behaviours associated with each NTS element. If you have the same element listed more than once then you will have to provide a separate example for each.

Non-Technical Skills Bingo

Dieckmann et al, Advances in Simulation (2016). DOI: 10.1186/s41077-016-0024-z

|  | Card 36 |
| --- | --- |
| 1 | Gathering information |
|  |  |
| 2 | Coordinating activities |
|  |  |
| 3 | Prioritizing |
|  |  |
| 4 | Prioritizing |
|  |  |
| 5 | Supporting others |
|  |  |

https://www.regionh.dk/CAMES/Forskning/Forskningsprojekter/Sider/ANTSdk.aspx

# Instructions

Use this card for the movie scene that you will see. Take notes in the NTS element for which you saw a good or a bad behavioural example. Such behaviour can also be in relation to how the people acting in the scene interact with devices or other parts of The environment. When you have completed all the boxes with examples shout ‘Bingo’. You will then be asked to describe the behaviours associated with each NTS element. If you have the same element listed more than once then you will have to provide a separate example for each.

Non-Technical Skills Bingo

Dieckmann et al, Advances in Simulation (2016). DOI: 10.1186/s41077-016-0024-z

|  | **Card 37** |
| --- | --- |
| 1 | Recognising and understanding contexts |
|  |  |
| 2 | Using authority and assertiveness |
|  |  |
| 3 | Anticipating and thinking ahead |
|  |  |
| 4 | Coordinating activities |
|  |  |
| 5 | Using authority and assertiveness |
|  |  |

https://www.regionh.dk/CAMES/Forskning/Forskningsprojekter/Sider/ANTSdk.aspx

# Instructions

Use this card for the movie scene that you will see. Take notes in the NTS element for which you saw a good or a bad behavioural example. Such behaviour can also be in relation to how the people acting in the scene interact with devices or other parts of The environment. When you have completed all the boxes with examples shout ‘Bingo’. You will then be asked to describe the behaviours associated with each NTS element. If you have the same element listed more than once then you will have to provide a separate example for each.

Non-Technical Skills Bingo

Dieckmann et al, Advances in Simulation (2016). DOI: 10.1186/s41077-016-0024-z

|  | Card 38 |
| --- | --- |
| 1 | Anticipating and thinking ahead |
|  |  |
| 2 | Recognising and understanding contexts |
|  |  |
| 3 | Gathering information |
|  |  |
| 4 | Using authority and assertiveness |
|  |  |
| 5 | Anticipating and thinking ahead |
|  |  |

https://www.regionh.dk/CAMES/Forskning/Forskningsprojekter/Sider/ANTSdk.aspx

# Instructions

Use this card for the movie scene that you will see. Take notes in the NTS element for which you saw a good or a bad behavioural example. Such behaviour can also be in relation to how the people acting in the scene interact with devices or other parts of The environment. When you have completed all the boxes with examples shout ‘Bingo’. You will then be asked to describe the behaviours associated with each NTS element. If you have the same element listed more than once then you will have to provide a separate example for each.

Non-Technical Skills Bingo

Dieckmann et al, Advances in Simulation (2016). DOI: 10.1186/s41077-016-0024-z

|  | **Card 39** |
| --- | --- |
| 1 | Coordinating activities |
|  |  |
| 2 | Demonstrating self-awareness |
|  |  |
| 3 | Exchanging information |
|  |  |
| 4 | Demonstrating self-awareness |
|  |  |
| 5 | Identifying and utilizing resources |
|  |  |

https://www.regionh.dk/CAMES/Forskning/Forskningsprojekter/Sider/ANTSdk.aspx

# Instructions

Use this card for the movie scene that you will see. Take notes in the NTS element for which you saw a good or a bad behavioural example. Such behaviour can also be in relation to how the people acting in the scene interact with devices or other parts of The environment. When you have completed all the boxes with examples shout ‘Bingo’. You will then be asked to describe the behaviours associated with each NTS element. If you have the same element listed more than once then you will have to provide a separate example for each.

Non-Technical Skills Bingo

Dieckmann et al, Advances in Simulation (2016). DOI: 10.1186/s41077-016-0024-z

|  | Card 40 |
| --- | --- |
| 1 | Coordinating activities |
|  |  |
| 2 | Coordinating activities |
|  |  |
| 3 | Exchanging information |
|  |  |
| 4 | Demonstrating self-awareness |
|  |  |
| 5 | Recognising and understanding contexts |
|  |  |

https://www.regionh.dk/CAMES/Forskning/Forskningsprojekter/Sider/ANTSdk.aspx

# Instructions

Use this card for the movie scene that you will see. Take notes in the NTS element for which you saw a good or a bad behavioural example. Such behaviour can also be in relation to how the people acting in the scene interact with devices or other parts of The environment. When you have completed all the boxes with examples shout ‘Bingo’. You will then be asked to describe the behaviours associated with each NTS element. If you have the same element listed more than once then you will have to provide a separate example for each.

Non-Technical Skills Bingo

Dieckmann et al, Advances in Simulation (2016). DOI: 10.1186/s41077-016-0024-z

|  | **Card 41** |
| --- | --- |
| 1 | Identifying and utilizing resources |
|  |  |
| 2 | Identifying and utilizing resources |
|  |  |
| 3 | Anticipating and thinking ahead |
|  |  |
| 4 | Recognising and understanding contexts |
|  |  |
| 5 | Identifying and utilizing resources |
|  |  |

https://www.regionh.dk/CAMES/Forskning/Forskningsprojekter/Sider/ANTSdk.aspx

# Instructions

Use this card for the movie scene that you will see. Take notes in the NTS element for which you saw a good or a bad behavioural example. Such behaviour can also be in relation to how the people acting in the scene interact with devices or other parts of The environment. When you have completed all the boxes with examples shout ‘Bingo’. You will then be asked to describe the behaviours associated with each NTS element. If you have the same element listed more than once then you will have to provide a separate example for each.

Non-Technical Skills Bingo

Dieckmann et al, Advances in Simulation (2016). DOI: 10.1186/s41077-016-0024-z

|  | Card 42 |
| --- | --- |
| 1 | Identifying and utilizing resources |
|  |  |
| 2 | Recognising and understanding contexts |
|  |  |
| 3 | Anticipating and thinking ahead |
|  |  |
| 4 | Identifying options |
|  |  |
| 5 | Choosing, communicating and implementing decisions |
|  |  |

https://www.regionh.dk/CAMES/Forskning/Forskningsprojekter/Sider/ANTSdk.aspx

# Instructions

Use this card for the movie scene that you will see. Take notes in the NTS element for which you saw a good or a bad behavioural example. Such behaviour can also be in relation to how the people acting in the scene interact with devices or other parts of The environment. When you have completed all the boxes with examples shout ‘Bingo’. You will then be asked to describe the behaviours associated with each NTS element. If you have the same element listed more than once then you will have to provide a separate example for each.

Non-Technical Skills Bingo

Dieckmann et al, Advances in Simulation (2016). DOI: 10.1186/s41077-016-0024-z

|  | **Card 43** |
| --- | --- |
| 1 | Choosing, communicating and implementing decisions |
|  |  |
| 2 | Supporting others |
|  |  |
| 3 | Assessing Competencies |
|  |  |
| 4 | Assessing Competencies |
|  |  |
| 5 | Identifying and utilizing resources |
|  |  |

https://www.regionh.dk/CAMES/Forskning/Forskningsprojekter/Sider/ANTSdk.aspx

# Instructions

Use this card for the movie scene that you will see. Take notes in the NTS element for which you saw a good or a bad behavioural example. Such behaviour can also be in relation to how the people acting in the scene interact with devices or other parts of The environment. When you have completed all the boxes with examples shout ‘Bingo’. You will then be asked to describe the behaviours associated with each NTS element. If you have the same element listed more than once then you will have to provide a separate example for each.

Non-Technical Skills Bingo

Dieckmann et al, Advances in Simulation (2016). DOI: 10.1186/s41077-016-0024-z

|  | Card 44 |
| --- | --- |
| 1 | Anticipating and thinking ahead |
|  |  |
| 2 | Planning and preparing |
|  |  |
| 3 | Anticipating and thinking ahead |
|  |  |
| 4 | Reassessing decisions |
|  |  |
| 5 | Choosing, communicating and implementing decisions |
|  |  |

https://www.regionh.dk/CAMES/Forskning/Forskningsprojekter/Sider/ANTSdk.aspx

# Instructions

Use this card for the movie scene that you will see. Take notes in the NTS element for which you saw a good or a bad behavioural example. Such behaviour can also be in relation to how the people acting in the scene interact with devices or other parts of The environment. When you have completed all the boxes with examples shout ‘Bingo’. You will then be asked to describe the behaviours associated with each NTS element. If you have the same element listed more than once then you will have to provide a separate example for each.

Non-Technical Skills Bingo

Dieckmann et al, Advances in Simulation (2016). DOI: 10.1186/s41077-016-0024-z

|  | **Card 45** |
| --- | --- |
| 1 | Gathering information |
|  |  |
| 2 | Coordinating activities |
|  |  |
| 3 | Reassessing decisions |
|  |  |
| 4 | Demonstrating self-awareness |
|  |  |
| 5 | Identifying and utilizing resources |
|  |  |

https://www.regionh.dk/CAMES/Forskning/Forskningsprojekter/Sider/ANTSdk.aspx

# Instructions

Use this card for the movie scene that you will see. Take notes in the NTS element for which you saw a good or a bad behavioural example. Such behaviour can also be in relation to how the people acting in the scene interact with devices or other parts of The environment. When you have completed all the boxes with examples shout ‘Bingo’. You will then be asked to describe the behaviours associated with each NTS element. If you have the same element listed more than once then you will have to provide a separate example for each.

Non-Technical Skills Bingo

Dieckmann et al, Advances in Simulation (2016). DOI: 10.1186/s41077-016-0024-z

|  | Card 46 |
| --- | --- |
| 1 | Reassessing decisions |
|  |  |
| 2 | Reassessing decisions |
|  |  |
| 3 | Recognising and understanding contexts |
|  |  |
| 4 | Prioritizing |
|  |  |
| 5 | Identifying and utilizing resources |
|  |  |

https://www.regionh.dk/CAMES/Forskning/Forskningsprojekter/Sider/ANTSdk.aspx

# Instructions

Use this card for the movie scene that you will see. Take notes in the NTS element for which you saw a good or a bad behavioural example. Such behaviour can also be in relation to how the people acting in the scene interact with devices or other parts of The environment. When you have completed all the boxes with examples shout ‘Bingo’. You will then be asked to describe the behaviours associated with each NTS element. If you have the same element listed more than once then you will have to provide a separate example for each.

Non-Technical Skills Bingo

Dieckmann et al, Advances in Simulation (2016). DOI: 10.1186/s41077-016-0024-z

|  | **Card 47** |
| --- | --- |
| 1 | Identifying options |
|  |  |
| 2 | Reassessing decisions |
|  |  |
| 3 | Planning and preparing |
|  |  |
| 4 | Providing and maintaining standards |
|  |  |
| 5 | Identifying and utilizing resources |
|  |  |

https://www.regionh.dk/CAMES/Forskning/Forskningsprojekter/Sider/ANTSdk.aspx

# Instructions

Use this card for the movie scene that you will see. Take notes in the NTS element for which you saw a good or a bad behavioural example. Such behaviour can also be in relation to how the people acting in the scene interact with devices or other parts of The environment. When you have completed all the boxes with examples shout ‘Bingo’. You will then be asked to describe the behaviours associated with each NTS element. If you have the same element listed more than once then you will have to provide a separate example for each.

Non-Technical Skills Bingo

Dieckmann et al, Advances in Simulation (2016). DOI: 10.1186/s41077-016-0024-z

|  | Card 48 |
| --- | --- |
| 1 | Choosing, communicating and implementing decisions |
|  |  |
| 2 | Supporting others |
|  |  |
| 3 | Planning and preparing |
|  |  |
| 4 | Reassessing decisions |
|  |  |
| 5 | Reassessing decisions |
|  |  |

https://www.regionh.dk/CAMES/Forskning/Forskningsprojekter/Sider/ANTSdk.aspx

# Instructions

Use this card for the movie scene that you will see. Take notes in the NTS element for which you saw a good or a bad behavioural example. Such behaviour can also be in relation to how the people acting in the scene interact with devices or other parts of The environment. When you have completed all the boxes with examples shout ‘Bingo’. You will then be asked to describe the behaviours associated with each NTS element. If you have the same element listed more than once then you will have to provide a separate example for each.

Non-Technical Skills Bingo

Dieckmann et al, Advances in Simulation (2016). DOI: 10.1186/s41077-016-0024-z

|  | **Card 49** |
| --- | --- |
| 1 | Demonstrating self-awareness |
|  |  |
| 2 | Identifying and utilizing resources |
|  |  |
| 3 | Supporting others |
|  |  |
| 4 | Reassessing decisions |
|  |  |
| 5 | Supporting others |
|  |  |

https://www.regionh.dk/CAMES/Forskning/Forskningsprojekter/Sider/ANTSdk.aspx

# Instructions

Use this card for the movie scene that you will see. Take notes in the NTS element for which you saw a good or a bad behavioural example. Such behaviour can also be in relation to how the people acting in the scene interact with devices or other parts of The environment. When you have completed all the boxes with examples shout ‘Bingo’. You will then be asked to describe the behaviours associated with each NTS element. If you have the same element listed more than once then you will have to provide a separate example for each.

Non-Technical Skills Bingo

Dieckmann et al, Advances in Simulation (2016). DOI: 10.1186/s41077-016-0024-z

|  | Card 50 |
| --- | --- |
| 1 | Recognising and understanding contexts |
|  |  |
| 2 | Demonstrating self-awareness |
|  |  |
| 3 | Demonstrating self-awareness |
|  |  |
| 4 | Identifying options |
|  |  |
| 5 | Assessing Competencies |
|  |  |

https://www.regionh.dk/CAMES/Forskning/Forskningsprojekter/Sider/ANTSdk.aspx

# Instructions

Use this card for the movie scene that you will see. Take notes in the NTS element for which you saw a good or a bad behavioural example. Such behaviour can also be in relation to how the people acting in the scene interact with devices or other parts of The environment. When you have completed all the boxes with examples shout ‘Bingo’. You will then be asked to describe the behaviours associated with each NTS element. If you have the same element listed more than once then you will have to provide a separate example for each.

Non-Technical Skills Bingo

Dieckmann et al, Advances in Simulation (2016). DOI: 10.1186/s41077-016-0024-z

|  | **Card 51** |
| --- | --- |
| 1 | Recognising and understanding contexts |
|  |  |
| 2 | Identifying options |
|  |  |
| 3 | Prioritizing |
|  |  |
| 4 | Choosing, communicating and implementing decisions |
|  |  |
| 5 | Using authority and assertiveness |
|  |  |

https://www.regionh.dk/CAMES/Forskning/Forskningsprojekter/Sider/ANTSdk.aspx

# Instructions

Use this card for the movie scene that you will see. Take notes in the NTS element for which you saw a good or a bad behavioural example. Such behaviour can also be in relation to how the people acting in the scene interact with devices or other parts of The environment. When you have completed all the boxes with examples shout ‘Bingo’. You will then be asked to describe the behaviours associated with each NTS element. If you have the same element listed more than once then you will have to provide a separate example for each.

Non-Technical Skills Bingo

Dieckmann et al, Advances in Simulation (2016). DOI: 10.1186/s41077-016-0024-z

|  | Card 52 |
| --- | --- |
| 1 | Anticipating and thinking ahead |
|  |  |
| 2 | Anticipating and thinking ahead |
|  |  |
| 3 | Using authority and assertiveness |
|  |  |
| 4 | Gathering information |
|  |  |
| 5 | Planning and preparing |
|  |  |

https://www.regionh.dk/CAMES/Forskning/Forskningsprojekter/Sider/ANTSdk.aspx

# Instructions

Use this card for the movie scene that you will see. Take notes in the NTS element for which you saw a good or a bad behavioural example. Such behaviour can also be in relation to how the people acting in the scene interact with devices or other parts of The environment. When you have completed all the boxes with examples shout ‘Bingo’. You will then be asked to describe the behaviours associated with each NTS element. If you have the same element listed more than once then you will have to provide a separate example for each.

Non-Technical Skills Bingo

Dieckmann et al, Advances in Simulation (2016). DOI: 10.1186/s41077-016-0024-z

|  | **Card 53** |
| --- | --- |
| 1 | Exchanging information |
|  |  |
| 2 | Supporting others |
|  |  |
| 3 | Planning and preparing |
|  |  |
| 4 | Gathering information |
|  |  |
| 5 | Assessing Competencies |
|  |  |

https://www.regionh.dk/CAMES/Forskning/Forskningsprojekter/Sider/ANTSdk.aspx

# Instructions

Use this card for the movie scene that you will see. Take notes in the NTS element for which you saw a good or a bad behavioural example. Such behaviour can also be in relation to how the people acting in the scene interact with devices or other parts of The environment. When you have completed all the boxes with examples shout ‘Bingo’. You will then be asked to describe the behaviours associated with each NTS element. If you have the same element listed more than once then you will have to provide a separate example for each.

Non-Technical Skills Bingo

Dieckmann et al, Advances in Simulation (2016). DOI: 10.1186/s41077-016-0024-z

|  | Card 54 |
| --- | --- |
| 1 | Supporting others |
|  |  |
| 2 | Identifying options |
|  |  |
| 3 | Choosing, communicating and implementing decisions |
|  |  |
| 4 | Exchanging information |
|  |  |
| 5 | Demonstrating self-awareness |
|  |  |

https://www.regionh.dk/CAMES/Forskning/Forskningsprojekter/Sider/ANTSdk.aspx

# Instructions

Use this card for the movie scene that you will see. Take notes in the NTS element for which you saw a good or a bad behavioural example. Such behaviour can also be in relation to how the people acting in the scene interact with devices or other parts of The environment. When you have completed all the boxes with examples shout ‘Bingo’. You will then be asked to describe the behaviours associated with each NTS element. If you have the same element listed more than once then you will have to provide a separate example for each.

Non-Technical Skills Bingo

Dieckmann et al, Advances in Simulation (2016). DOI: 10.1186/s41077-016-0024-z

|  | **Card 55** |
| --- | --- |
| 1 | Using authority and assertiveness |
|  |  |
| 2 | Coordinating activities |
|  |  |
| 3 | Planning and preparing |
|  |  |
| 4 | Using authority and assertiveness |
|  |  |
| 5 | Prioritizing |
|  |  |

https://www.regionh.dk/CAMES/Forskning/Forskningsprojekter/Sider/ANTSdk.aspx

# Instructions

Use this card for the movie scene that you will see. Take notes in the NTS element for which you saw a good or a bad behavioural example. Such behaviour can also be in relation to how the people acting in the scene interact with devices or other parts of The environment. When you have completed all the boxes with examples shout ‘Bingo’. You will then be asked to describe the behaviours associated with each NTS element. If you have the same element listed more than once then you will have to provide a separate example for each.

Non-Technical Skills Bingo

Dieckmann et al, Advances in Simulation (2016). DOI: 10.1186/s41077-016-0024-z

|  | Card 56 |
| --- | --- |
| 1 | Assessing Competencies |
|  |  |
| 2 | Identifying options |
|  |  |
| 3 | Planning and preparing |
|  |  |
| 4 | Choosing, communicating and implementing decisions |
|  |  |
| 5 | Reassessing decisions |
|  |  |

https://www.regionh.dk/CAMES/Forskning/Forskningsprojekter/Sider/ANTSdk.aspx

# Instructions

Use this card for the movie scene that you will see. Take notes in the NTS element for which you saw a good or a bad behavioural example. Such behaviour can also be in relation to how the people acting in the scene interact with devices or other parts of The environment. When you have completed all the boxes with examples shout ‘Bingo’. You will then be asked to describe the behaviours associated with each NTS element. If you have the same element listed more than once then you will have to provide a separate example for each.

Non-Technical Skills Bingo

Dieckmann et al, Advances in Simulation (2016). DOI: 10.1186/s41077-016-0024-z

|  | **Card 57** |
| --- | --- |
| 1 | Identifying options |
|  |  |
| 2 | Providing and maintaining standards |
|  |  |
| 3 | Coordinating activities |
|  |  |
| 4 | Exchanging information |
|  |  |
| 5 | Planning and preparing |
|  |  |

https://www.regionh.dk/CAMES/Forskning/Forskningsprojekter/Sider/ANTSdk.aspx

# Instructions

Use this card for the movie scene that you will see. Take notes in the NTS element for which you saw a good or a bad behavioural example. Such behaviour can also be in relation to how the people acting in the scene interact with devices or other parts of The environment. When you have completed all the boxes with examples shout ‘Bingo’. You will then be asked to describe the behaviours associated with each NTS element. If you have the same element listed more than once then you will have to provide a separate example for each.

Non-Technical Skills Bingo

Dieckmann et al, Advances in Simulation (2016). DOI: 10.1186/s41077-016-0024-z

|  | Card 58 |
| --- | --- |
| 1 | Identifying options |
|  |  |
| 2 | Coordinating activities |
|  |  |
| 3 | Using authority and assertiveness |
|  |  |
| 4 | Identifying and utilizing resources |
|  |  |
| 5 | Gathering information |
|  |  |

https://www.regionh.dk/CAMES/Forskning/Forskningsprojekter/Sider/ANTSdk.aspx

# Instructions

Use this card for the movie scene that you will see. Take notes in the NTS element for which you saw a good or a bad behavioural example. Such behaviour can also be in relation to how the people acting in the scene interact with devices or other parts of The environment. When you have completed all the boxes with examples shout ‘Bingo’. You will then be asked to describe the behaviours associated with each NTS element. If you have the same element listed more than once then you will have to provide a separate example for each.

Non-Technical Skills Bingo

Dieckmann et al, Advances in Simulation (2016). DOI: 10.1186/s41077-016-0024-z

|  | **Card 59** |
| --- | --- |
| 1 | Demonstrating self-awareness |
|  |  |
| 2 | Reassessing decisions |
|  |  |
| 3 | Using authority and assertiveness |
|  |  |
| 4 | Supporting others |
|  |  |
| 5 | Demonstrating self-awareness |
|  |  |

https://www.regionh.dk/CAMES/Forskning/Forskningsprojekter/Sider/ANTSdk.aspx

# Instructions

Use this card for the movie scene that you will see. Take notes in the NTS element for which you saw a good or a bad behavioural example. Such behaviour can also be in relation to how the people acting in the scene interact with devices or other parts of The environment. When you have completed all the boxes with examples shout ‘Bingo’. You will then be asked to describe the behaviours associated with each NTS element. If you have the same element listed more than once then you will have to provide a separate example for each.

Non-Technical Skills Bingo

Dieckmann et al, Advances in Simulation (2016). DOI: 10.1186/s41077-016-0024-z

|  | Card 60 |
| --- | --- |
| 1 | Supporting others |
|  |  |
| 2 | Prioritizing |
|  |  |
| 3 | Anticipating and thinking ahead |
|  |  |
| 4 | Assessing Competencies |
|  |  |
| 5 | Demonstrating self-awareness |
|  |  |

https://www.regionh.dk/CAMES/Forskning/Forskningsprojekter/Sider/ANTSdk.aspx

# Instructions

Use this card for the movie scene that you will see. Take notes in the NTS element for which you saw a good or a bad behavioural example. Such behaviour can also be in relation to how the people acting in the scene interact with devices or other parts of The environment. When you have completed all the boxes with examples shout ‘Bingo’. You will then be asked to describe the behaviours associated with each NTS element. If you have the same element listed more than once then you will have to provide a separate example for each.

Non-Technical Skills Bingo

Dieckmann et al, Advances in Simulation (2016). DOI: 10.1186/s41077-016-0024-z

|  | **Card 61** |
| --- | --- |
| 1 | Assessing Competencies |
|  |  |
| 2 | Supporting others |
|  |  |
| 3 | Providing and maintaining standards |
|  |  |
| 4 | Recognising and understanding contexts |
|  |  |
| 5 | Coordinating activities |
|  |  |

https://www.regionh.dk/CAMES/Forskning/Forskningsprojekter/Sider/ANTSdk.aspx

# Instructions

Use this card for the movie scene that you will see. Take notes in the NTS element for which you saw a good or a bad behavioural example. Such behaviour can also be in relation to how the people acting in the scene interact with devices or other parts of The environment. When you have completed all the boxes with examples shout ‘Bingo’. You will then be asked to describe the behaviours associated with each NTS element. If you have the same element listed more than once then you will have to provide a separate example for each.

Non-Technical Skills Bingo

Dieckmann et al, Advances in Simulation (2016). DOI: 10.1186/s41077-016-0024-z

|  | Card 62 |
| --- | --- |
| 1 | Prioritizing |
|  |  |
| 2 | Identifying options |
|  |  |
| 3 | Identifying and utilizing resources |
|  |  |
| 4 | Assessing Competencies |
|  |  |
| 5 | Coordinating activities |
|  |  |

https://www.regionh.dk/CAMES/Forskning/Forskningsprojekter/Sider/ANTSdk.aspx

# Instructions

Use this card for the movie scene that you will see. Take notes in the NTS element for which you saw a good or a bad behavioural example. Such behaviour can also be in relation to how the people acting in the scene interact with devices or other parts of The environment. When you have completed all the boxes with examples shout ‘Bingo’. You will then be asked to describe the behaviours associated with each NTS element. If you have the same element listed more than once then you will have to provide a separate example for each.

Non-Technical Skills Bingo

Dieckmann et al, Advances in Simulation (2016). DOI: 10.1186/s41077-016-0024-z

|  | **Card 63** |
| --- | --- |
| 1 | Supporting others |
|  |  |
| 2 | Providing and maintaining standards |
|  |  |
| 3 | Using authority and assertiveness |
|  |  |
| 4 | Supporting others |
|  |  |
| 5 | Prioritizing |
|  |  |

https://www.regionh.dk/CAMES/Forskning/Forskningsprojekter/Sider/ANTSdk.aspx

# Instructions

Use this card for the movie scene that you will see. Take notes in the NTS element for which you saw a good or a bad behavioural example. Such behaviour can also be in relation to how the people acting in the scene interact with devices or other parts of The environment. When you have completed all the boxes with examples shout ‘Bingo’. You will then be asked to describe the behaviours associated with each NTS element. If you have the same element listed more than once then you will have to provide a separate example for each.

Non-Technical Skills Bingo

Dieckmann et al, Advances in Simulation (2016). DOI: 10.1186/s41077-016-0024-z

|  | Card |
| --- | --- |
| 1 |  |
|  |  |
| 2 |  |
|  |  |
| 3 |  |
|  |  |
| 4 |  |
|  |  |
| 5 |  |
|  |  |

https://www.regionh.dk/CAMES/Forskning/Forskningsprojekter/Sider/ANTSdk.aspx

# Instructions

Use this card for the movie scene that you will see. Take notes in the NTS element for which you saw a good or a bad behavioural example. Such behaviour can also be in relation to how the people acting in the scene interact with devices or other parts of The environment. When you have completed all the boxes with examples shout ‘Bingo’. You will then be asked to describe the behaviours associated with each NTS element. If you have the same element listed more than once then you will have to provide a separate example for each.
